# Supplementary material for: Asymmetric division events promote variability in cell cycle duration in animal cells and Escherichia coli
Source: Nat Commun. 2019 Apr 23;10:1901. doi: 10.1038/s41467-019-09413-5 (PMC6478688; doi:10.1038/s41467-019-09413-5)
Supplement: Supplementary file 1 — Supplementary Information [file 41467_2019_9413_MOESM1_ESM.pdf]

Supplementary information

to manuscript “Asymmetric division events promote variability in cell cycle duration in animal cells and *Escherichia coli*” by Berge U et al.

Supplementary figures

Supplementary Figure 1

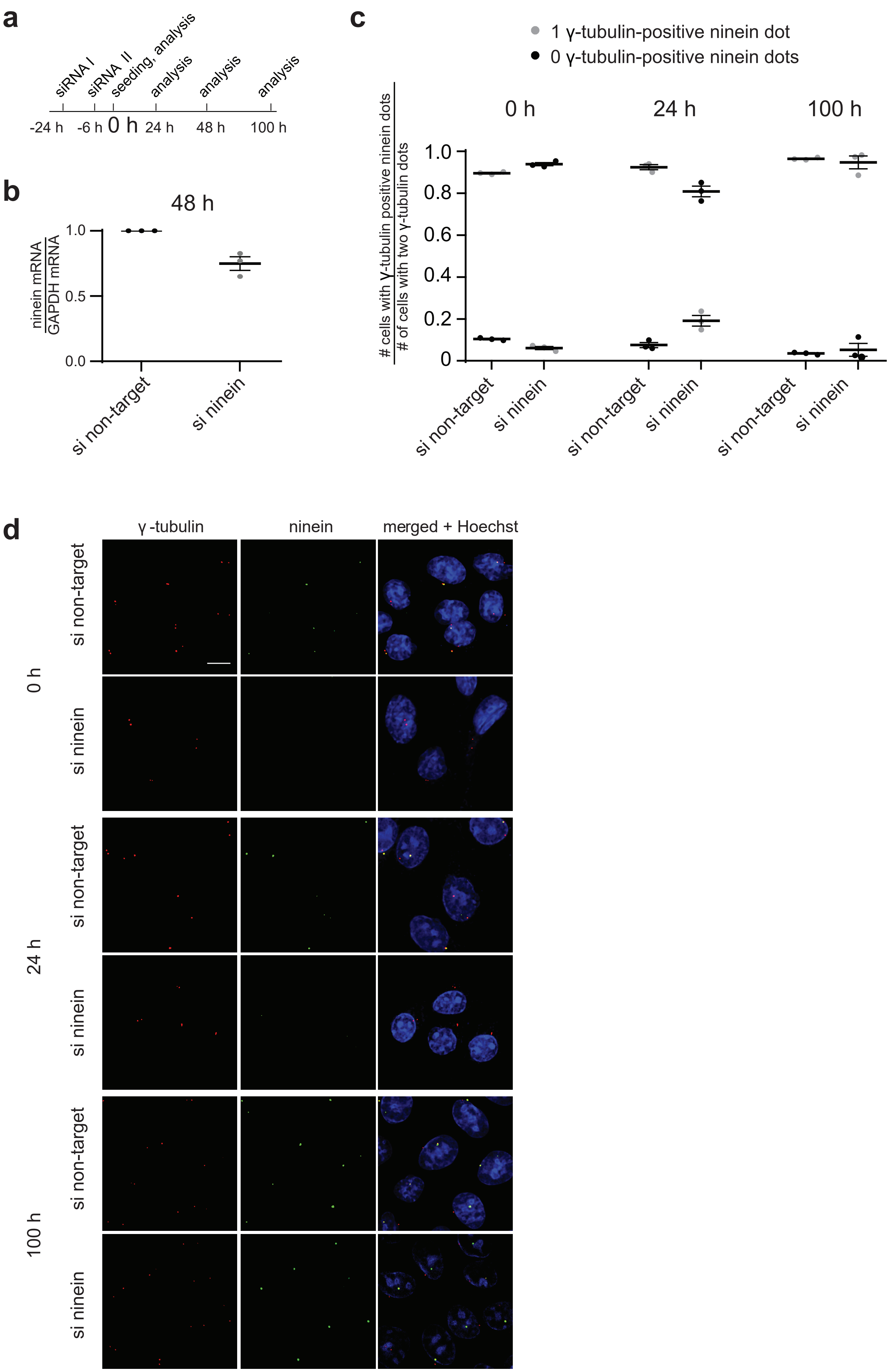

Supplementary Figure 1: Ninein downregulation efficiency.

a) Experimental scheme indicating the temporal sequence in panel b), c), d) of this figure. RNA I, II; two RNAi transfections before seeding the cells.

b) Cells transfected with siRNA against a mix of 4 oligonucleotides against ninein have 88% reduced ninein mRNA levels compared to the non-target treatment using a commercial pool of non-target siRNAs. Quantification of ninein mRNA levels by real-time RT-PCR 48 h after seeding for analysis. Threshold cycle times (Ct) were normalised to the Ct of GAPDH mRNA. Data point – single experiment; central line - mean; whiskers - s.e.m..

c) Quantification of ninein downregulation by immunofluorescence (example images in d). Quantification of ninein-positive centrosomes (labelled with γ-tubulin) in interphase cells. Between 100 and 116 cells were analysed per condition and experiment. Data point – single experiment; central line - mean; whiskers - s.e.m..

d) Representative immunofluorescence images of si non-target and si ninein cells underlying the quantification shown in c). Images were acquired using the same microscope exposure settings. c, d) The cells labelled for ninein (green), γ-tubulin (red) and nuclei (blue) show a lack of centrosomal staining in depleted cells at 0 h (94% +/- 0.88%) and 24 h (80.7% +/- 1.76%) but not at 100 h (5.1% +/- 3.18%) after seeding for analysis.

Supplementary Figure 2

a Lineage tree

Generation of a lineage tree:
i) isolated attached single cells were selected on the microscope stage,
ii) their progeny imaged, and
iii) for each single selected cell a movie generated, which was used to track the individual cells, and
iv) one lineage tree (termed: lineage in the text) was generated from a single selected cell.
Here for example (corresponding to Figure 1a):

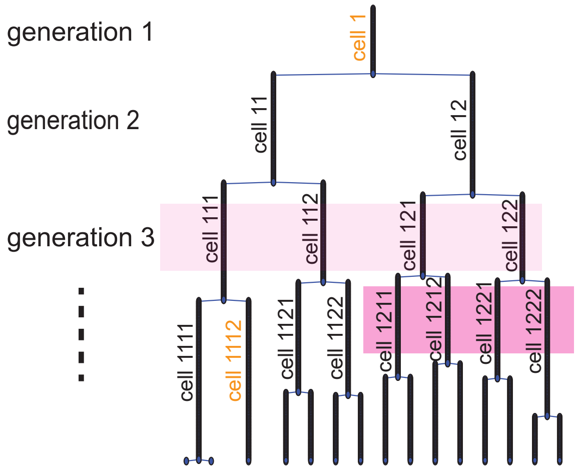

Cell cycle duration (ccd) definition:
imaged lifetime of a cell from end of a mitosis that generated the cell until the next mitosis, where two daughter cells are generated
Illustration of ccd in a lineage tree:
ccd corresponds to length of the vertical lines only if imaging covered the complete lifetime of a cell i.e. mitosis to mitosis (horizontal line).
Here: The ccds of cell1 and cell 1112 are therefore not available.

A granddaughter set is composed of 4 cell cycle durations corresponding to the four granddaugher cells (see b)
Here:
- 2 granddaughter sets of ccds (pink background: light, darker) are highlighted.
- Detail: As cell 1122 didn't divide before imaging stopped its ccd is not available.Therefore cell 1112, and its related granddaughter cells, the cell 1111, cell 1121 and cell 1122, do not constitute a granddaughter set.

b Lineage tree part

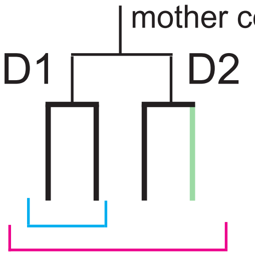

mother cell (e.g. cell 1 or cell 122 in lineage tree of a), incomplete list))

daughter cells (D1, D2, a pair of daughter cells is generated from a mother cell; D1 is sibling to D2. Generally: one division generates two sibling cells.

2 cousin cells of e.g. the green cell, a reference cell; 2 cousin cells are also sibling cells, term "cousin cells" is not used in this study as reference cell dependent)

4 granddaughter cells
(originating from 2 daughter cells, which originate from the same mother cell)

c Relative outlier cells

Using the
Outlier Detection Method (ODM)
(see supplementary Figure S5)
only
RELATIVE outlying
cell cycle durations in
a granddaughter set
are detectable.

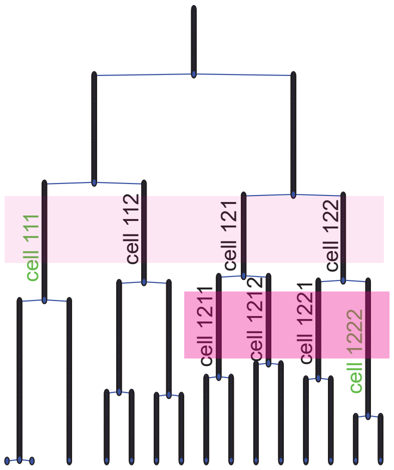

Here:
Two relative oulier cells in terms of their ccd:
cell 111
cell 1222
each cell is part of a distinct group of granddaughter cells

Here: In this lineage tree
the ccd of cell 111 is relative to the ccds of cell 112, cell 121 and cell 122 longer
AND: The ccd of cell 1222 is relative to the ccds of cell 1211, cell 1212 and cell 1221 longer

Supplementary Figure 2: Terminology overview.

Scheme illustrating details of a) lineage trees, b) lineage tree parts and c) relative properties of outlier cells. The lineage tree of Figure 1a is shown exemplary in a), and c).

Supplementary Figure 3

a

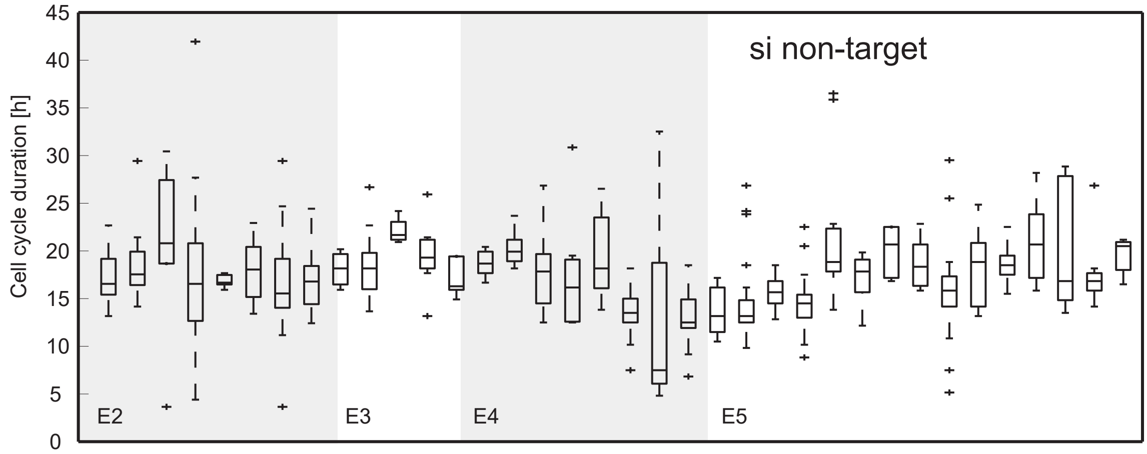

lineage ID	1	2	3	4	5	6	7	8	9	10	11	12	13	14	15	16	17	18	19	20	21	22	23	24	25	26	27	28	29	30	31	32	33	34	35	36
data points	14	8	6	28	5	14	24	26	3	13	4	8	6	6	6	8	11	5	24	15	27	14	30	14	19	15	5	6	8	15	10	14	10	6	9	5
max. generation	6	6	5	7	5	6	7	6	4	5	4	5	4	4	4	5	6	5	6	6	6	5	7	5	6	6	5	4	5	6	5	5	6	4	6	5
max. time [h]	68	58	63	76	76	76	76	76	76	76	62	80	71	80	80	67	73	85	63	73	73	51	69	53	64	84	84	84	84	66	82	70	82	58	84	83

b

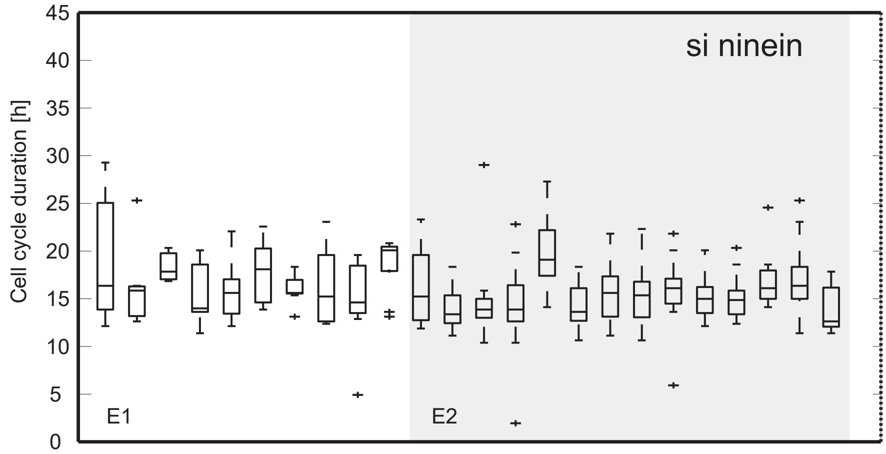

lineage ID	1	2	3	4	5	6	7	8	9	10	11	12	13	14	15	16	17	18	19	20	21	22	23	24
data points	6	7	5	6	11	9	9	6	12	9	24	31	15	17	7	23	14	13	12	16	14	7	24	9
max. generation	4	5	4	4	5	5	5	4	6	5	6	7	6	7	5	6	5	6	6	6	5	5	6	5
max. time [h]	64	64	64	64	64	64	64	64	64	64	76	76	76	68	76	65	65	65	65	65	65	76	71	57

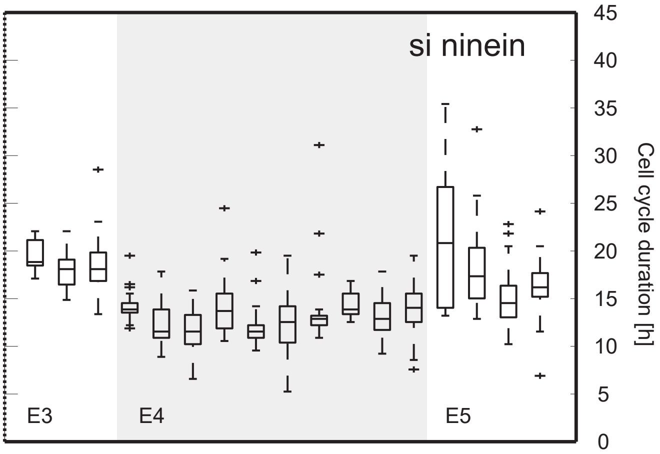

lineage ID	25	26	27	28	29	30	31	32	33	34	35	36	37	38	39	40	41
data points	9	8	10	29	22	21	22	70	28	18	12	28	22	9	18	43	13
max. generation	5	5	5	6	6	6	6	8	7	6	5	6	7	5	6	7	6
max. time [h]	80	80	80	66	58	60	71	85	63	67	56	63	72	80	84	84	84

Supplementary Figure 3: Median cell cycle durations as well as their variances differ more between individual lineages in si non-target than in si ninein MDCK cells.

a) Cell cycle duration distributions of individual lineages of si non-target treated cells of four independent experiments. Each boxplot represents the cell cycle duration distribution of individual cells within one lineage; central mark – median; edges of horizontal box – 1st and 3rd quartiles; whiskers - 1.5 times the IQR. The si non-target cells of experiment 1 (E1) did not qualify for movie analysis; E2 – E5, experiment 2 – experiment 5; for clarity plots of E2 and E4 have grey backgrounds. Below: lineage ID – unique identifier of analysed lineage tree; data points - number of cell cycle durations; max. generation – highest imaged generation rank of cells in given lineage tree; max. time - the last imaged time point. Distribution parameter ranges across all lineages of this condition: 7.3 h < medians < 21.5 h, Kruskal Wallis p = 6e-16; 0.5 h^2 < variance < 121 h^2; Levene's p = 7e-4.

b) Cell cycle duration distributions of individual lineage trees of si ninein cells. Display as in a) Distribution parameter ranges across all lineages of this condition: 11.7 h < medians < 21 h; Kruskal Wallis p = 4e-34; 1.9 h^2 < variance < 73.4 h^2, Levene's p < 1e-5; boxplots as in b). E1- E5 as in a) to demonstrate parallel imaging.

Supplementary Figure 4

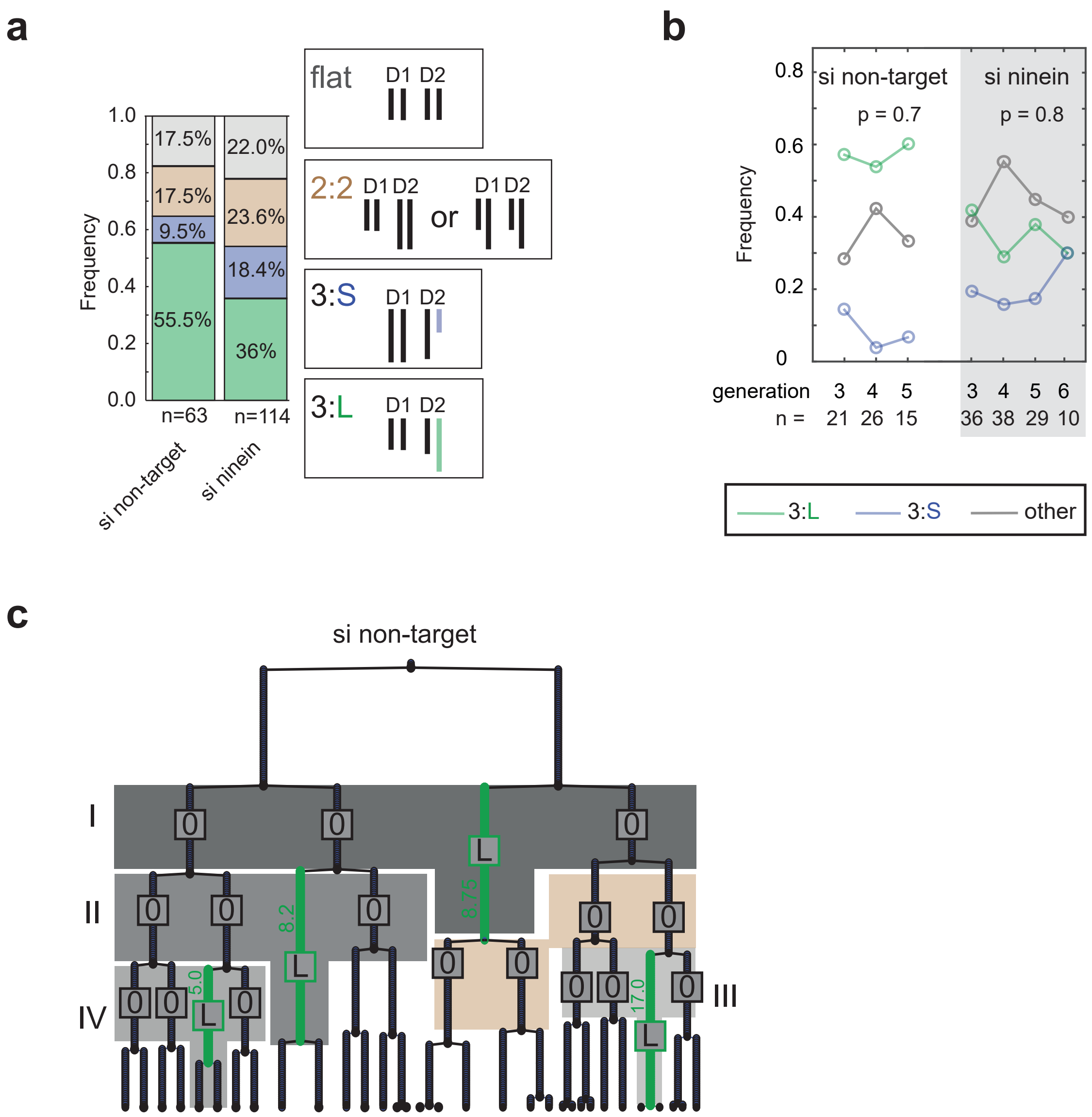

Supplementary Figure 4: Characterization of the diversity of cell cycle duration amongst granddaughter cells in si non-target and si ninein treated lineages.

a) The frequency of granddaughter sets exhibiting the 3:L- (green), 3:S- (blue), 2:2 (brown), and flat (grey) motif, respectively, based on the definition see below in Supplementary Figure 5 (threshold: dissimilarity index 2). Right, illustrations of the motifs by vertical lines representing the relative cell cycle durations of 4 granddaughter cells and a colour for a certain kind of outlier cell. D1, D2 are the two different daughter cells of the common mother cell. n - number of analysed granddaughter sets.

b) The frequency of 3:1 motifs per condition does not change significantly in the different generations. Si ninein data with grey background; two 3x3 Fisher's exact tests, p-values indicated; n - number of analysed granddaughter sets).

c) Illustration of 3:L and 2:2 motifs and corresponding L- and 0-cell classes in the si non-target lineage ID23 identified by our motif determination method (threshold: dissimilarity index 2). Four different 3:L motifs (granddaughter sets with grey backgrounds labelled I to IV), where one out of four related cells has a longer cell cycle duration (L-cell; green edge; vertical number reporting the corresponding dissimilarity index) relative to the other 3 (0-cells), are visually and by our quantitative outlier detection method (see below Supplementary Figure 5) detectable. One 2:2 motif (orange background) is also present, in which the cells have per definition a 0-cell identity assigned.

Supplementary Figure 5

Step 1
Sorting of granddaughter cell cycle durations with increasing values,
3 examples (3 differnt positions in a lineage tree indicated by cell ID):

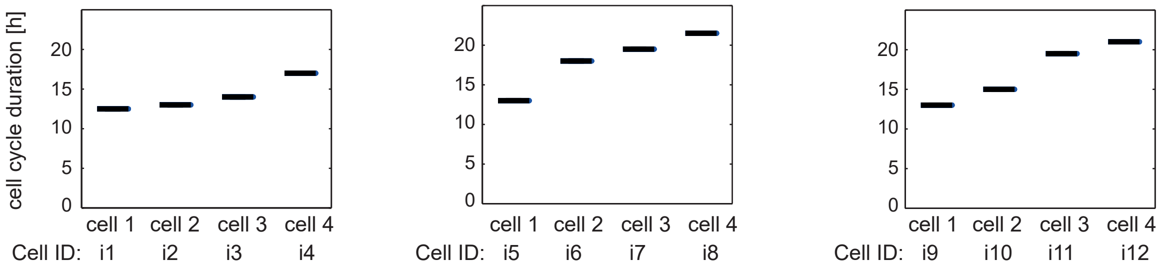

Step 2
All pairwise Mahalanobis distances for granddaughter sets
MD between cell z and cell y, with y and z being 1,2,3 or 4: $MD_{z\text{-}y} = 2 \times [(cell_z - mean(cell_z, cell_y))^2 / var(cell_1, cell_2, cell_3, cell_4)]^{0.5}$

cell z	cell y	MD	cell z	cell y	MD	cell z	cell y	MD
1	2	0.2481	1	2	1.3779	1	2	0.5333
1	3	0.7442	1	3	1.7913	1	3	1.7333
1	4	2.2326	1	4	2.3425	1	4	2.1333
2	3	0.4961	2	3	0.4134	2	3	1.2000
2	4	1.9846	2	4	0.9646	2	4	1.6000
3	4	1.4884	3	4	0.5512	3	4	0.4000

Step 3
Hierarchical clustering of granddaughter sets using single linkage

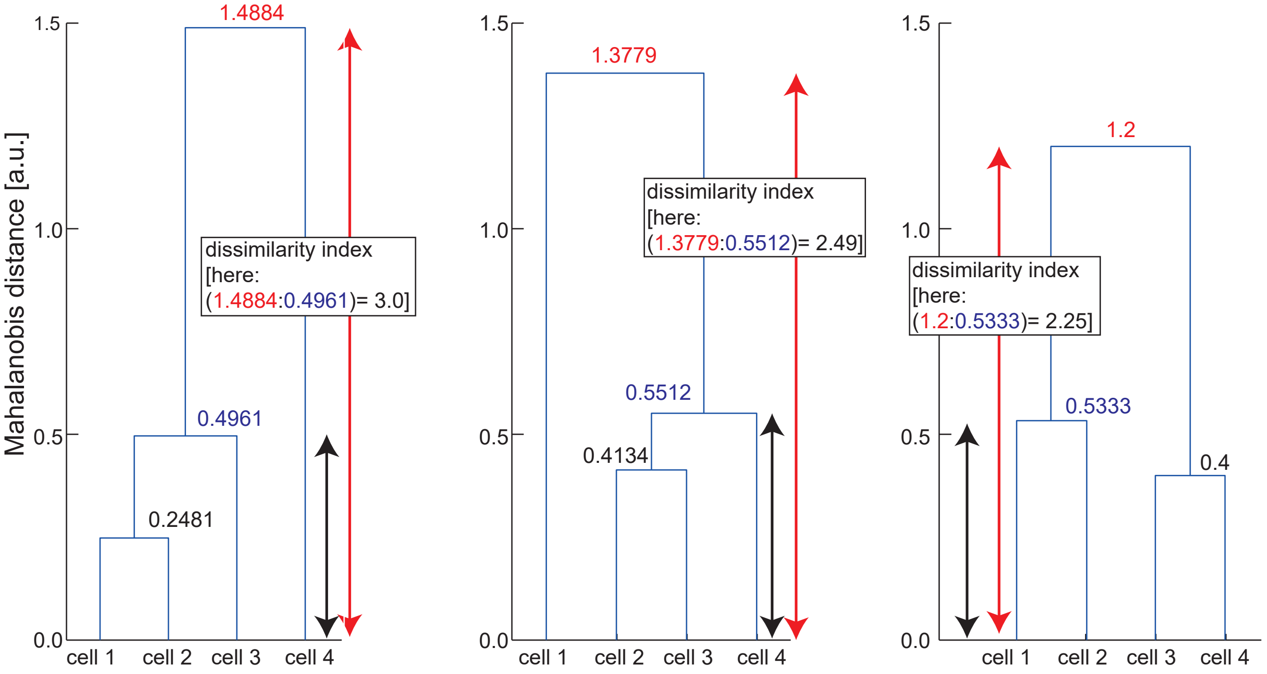

Step 4
Determination of motifs and cell classes for a defined dissimilarity index threshold, expressing the dissimilarity between the two most distant subclusters as their ratio (maximal/2nd hierarchy). A threshold of 2 indicates that the distance between the maximally linked and 2nd hierarchy subclusters is equal or larger than the sum of all distances within the rest data.

If the maximal linked MD involves
- cell 4 (longest cell cycle duration), then 3:L motif with cell 4 is L-cell, others 0-cells
- cell 1 (shortest cell cycle duration), then 3:S motif with cell 1 is S-cell, others 0-cells
- cell 2 and cell 3, then a 2:2 motif, and all cells are 0-cells
    - if cell 1 and 2 are X11/X12 and/or X21/X22, then siblings and thus 2:2 same daughter motif,
    - if cell 1 and 2 are X11/X21, X11/X22, X12/X21, and/or X12/X22, then cousins and 2:2 different daughter motif

else a “flat motif” is present and and all these granddaughter cells are 0-cells

Here motifs:

3:L	3:S	2:2

Supplementary Figure 5: Illustration of the outlier detection method (ODM).

The four steps are depicted with 3 exemplary granddaughter sets of cell cycle durations and lead to the determination of cell classes (L-, S-, and 0-cells). The individual cells of each granddaughter set are embedded in a lineage tree and therefore have unique cell IDs assigned, indicated only in step 1 as i1 – i12. In **step 1** the 4 cell cycle durations are ordered with increasing duration from left to right, assigned cell 1 to cell 4, respectively. In **step 2**, all pairwise Mahalanobis distances (MD) between the cell cycle durations are calculated. The smallest (black), 2nd smallest (blue), and 3rd smallest (red) distances were used to create the single linkage dendrogram of **step 3**. In **step 4**, X11, X12, X21, X22 represent cell IDs (see Figure 1a) with X being the cell ID of the common mother of each granddaughter set. Var, variance. This method is also usable to determine the frequency of any diversity motif, we only focus here on the outlier motifs and especially S- and L-cells.

Supplementary Figure 6

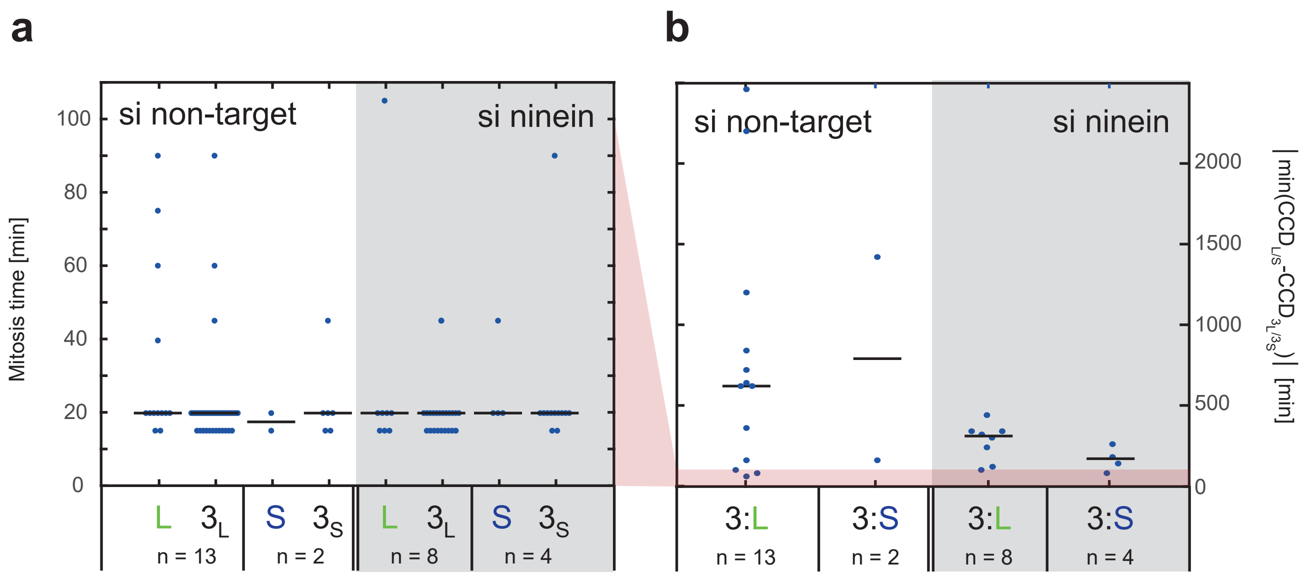

Supplementary Figure 6: Extremely long or short cell cycle durations are not due to aberrant mitotic durations in neither si non-target nor si ninein MDCK cells.

Cells with very extreme outlier cell cycle durations, where the outlier cell had minimally a 4x longer (L) or shorter (S) cell cycle duration than their three related granddaughter cells (3_L, $3_{S,}$ respectively) were chosen and the time of mitosis (based on the shape of the H2B fluorescence signal) measured. Si ninein data with grey background; n - frequency of analysed granddaughter set.

a) In the different cell classes of both non-target and ninein downregulated cells the mitotic durations did not differ (medians around 20 min, KW-test).

b) For each in a) assessed granddaughter sets, the minimal differences in cell cycle duration between the outlier cell and its corresponding 3 most related cells were plotted (medians from left to right: 620 min, 790 min, 310 min, 160 min). The red area illustrates the different maximal time scales of the graphs in a) and b).

Supplementary Figure 7

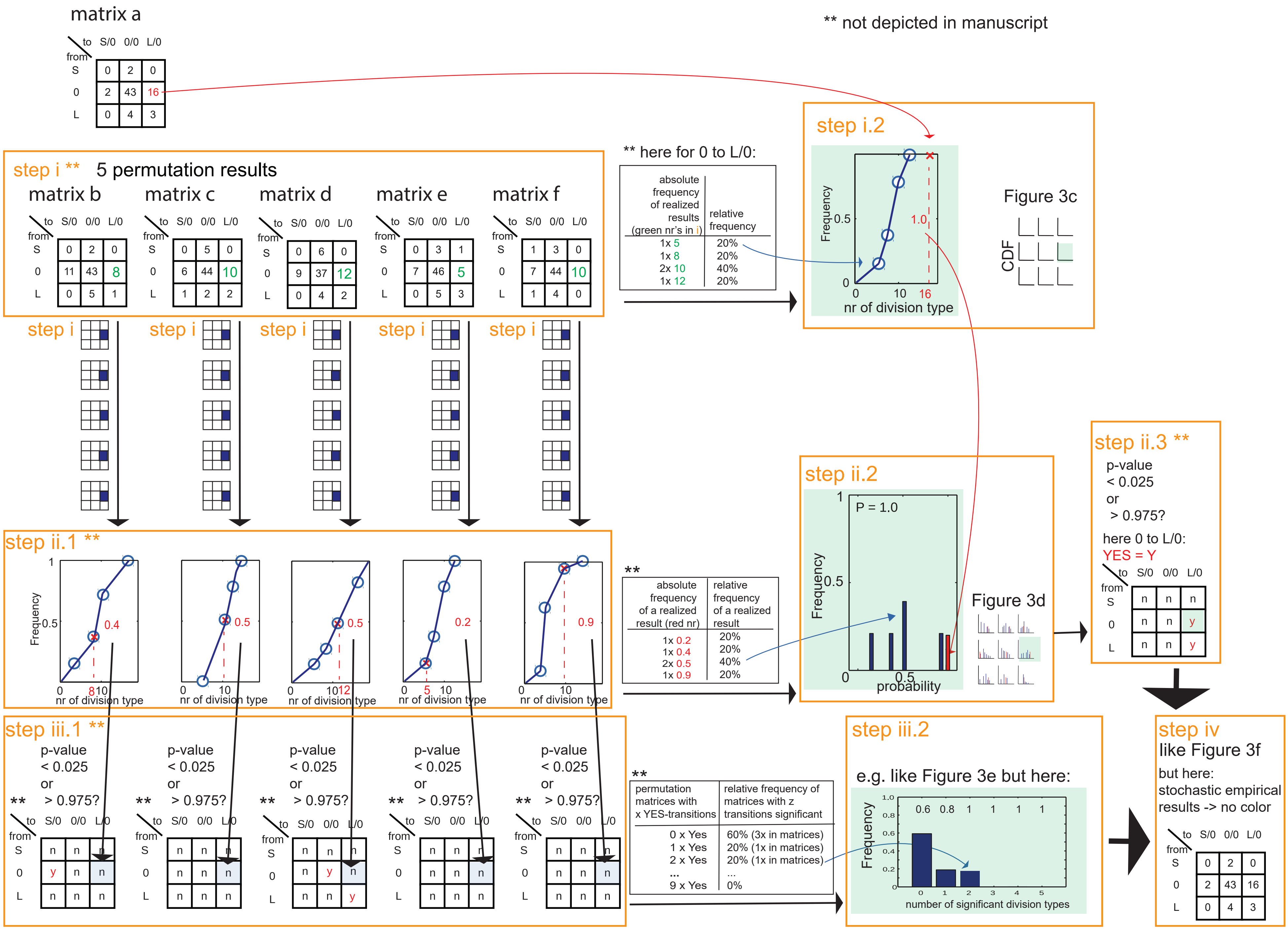

Supplementary Figure 7: Illustration of the propagation test

The null hypothesis of each propagation test is: the cell cycle durations in the daughter cells of any mother cell are not linked. The propagation test evaluates 1) which division type is within or outside the 95% confidence interval and 2) the probability to receive that number of significant division types by chance. The illustration is an invented scenario where one division type is significant (step ii) but the null model for the entire matrix is rejected (step iii).

For simplicity, here we focus on 5 permutations and the division type 0 to L/0. Green background signals reference to Figure 3. Arrows indicate links between the steps. Empirical reference Matrix a) to test the null hypothesis against.

Step i: Determination of the probability of obtaining the empirical division type with randomly permuted cell cycle durations. This step includes sequentially performing 1) the permutations (Figure 2b), 2) the ODM (Supplementary Figure 5), 3) establishment of a division type matrix as in Figure 3b and 4) in i.2 the generation of the CDF (Figure 3c).

Step ii: Permutation test: Each permutation is treated as the empirical one and plots as in i.2 are generated. The frequencies of those probabilities are plotted in step ii.2 as blue bars (e.g. Figure 3d). This distribution is compared to the empirical probability (step i.2, red) and interpreted (below, above, or within the 95% confidence interval) to result in step ii.3 - depicted as yes (y) and no (n) for significant and stochastic responses, respectively.

Step iii: Assessment of the power of the permutation test result. Step iii.1, each permutation of step ii.1 is evaluated regarding the 95% confidence interval. Step iii.2 represents the probability distribution of having 0, 1, 2, etc. significant division types (Figure 3e).

Step iv: Result of propagation test: Combination of two inputs: a) result of step ii.3 and b) of step iii.2. *Per definitionem*: Colour-code as in Figure 3f: only if two criteria are true: 1) x numbers of significant division types in step iii and 2) for x significant fields is the power-test (step iii) significant.

Supplementary Figure 8

a upper confidence limit

MDCK si non-target

from \ to	S/0	0/0	L/0
S	2	5	2
0	12	49	14
L	3	8	3

MDCK si ninein

from \ to	S/0	0/0	L/0
S	2	8	3
0	18	101	31
L	4	16	6

b expected value

MDCK si non-target

from \ to	S/0	0/0	L/0
S	0	2	0
0	8	42	10
L	1	5	1

MDCK si ninein

from \ to	S/0	0/0	L/0
S	1	4	1
0	13	90	24
L	1	11	3

c lower confidence limit

MDCK si non-target

from \ to	S/0	0/0	L/0
S	n.a.	n.a.	n.a.
0	4	35	6
L	n.a.	2	n.a.

MDCK si ninein

from \ to	S/0	0/0	L/0
S	n.a.	1	n.a.
0	8	80	17
L	n.a.	6	n.a.

d empirical

MDCK si non-target

from \ to	S/0	0/0	L/0
S	0	2	0
0	2	43	16
L	0	4	3

MDCK si ninein

from \ to	S/0	0/0	L/0
S	1	4	0
0	12	101	21
L	1	5	3

Supplementary Figure 8: Illustration of the shift of significant from non-significant division type frequencies.

Left panels: MDCK si non-target; right panels: MDCK si ninein.

a) Upper confidence limit (>97.5%; Figure 3c).

b) Expected values of 10,000 permutations (approx. 50% in Figure 3c).

c) Lower confidence limit (<2.5%, Figure 3c). Values equal or within the range of division type frequencies depicted in a and c are non-significant.

d) Empirical division type frequencies (Figures 3f, 4a, S9).

a-d) Colour code only for this figure: grey - clearly within the confidence interval of a random scenario; orange - upper confidence limit value; light green - lower confidence limit value; red - above the upper confidence limit value; dark green - below the lower confidence limit value; grey – expected value or n.a.. Of note, the number of fields outside the confidence interval is one of two inputs for the decision if the entire division type matrix is significant or not (Supplementary Figure 6); n.a. not applicable as no lower border, even with zero divisions.

Supplementary Figure 9

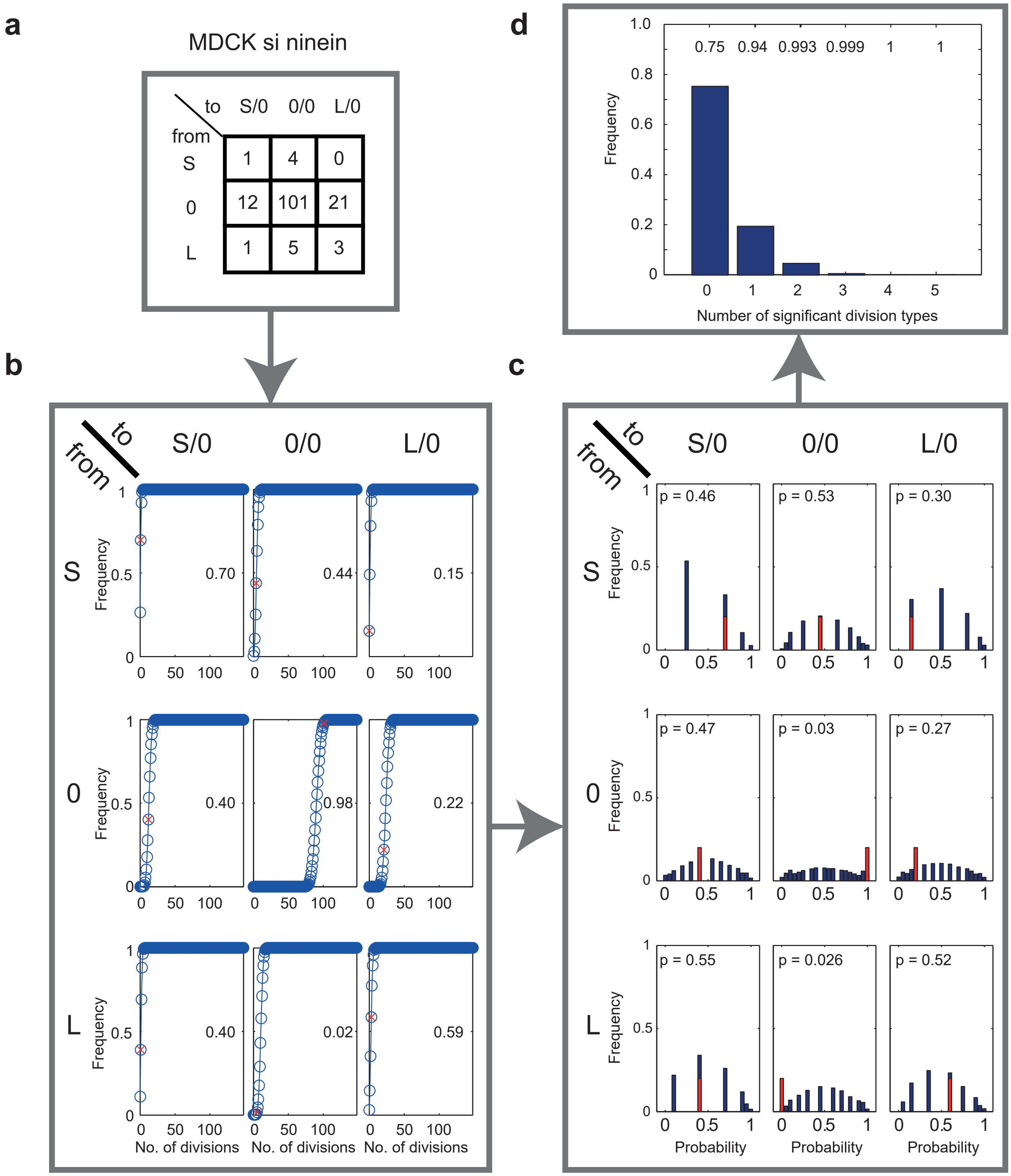

Supplementary Figure 9: Statistical propagation test for si ninein MDCK cells.

a) - d) Corresponds to description in Figure 3b-e but here for si ninein MDCK cells. The absolute and relative summary matrices are presented in Figure 4a and 5a, respectively.

Supplementary Figure 10

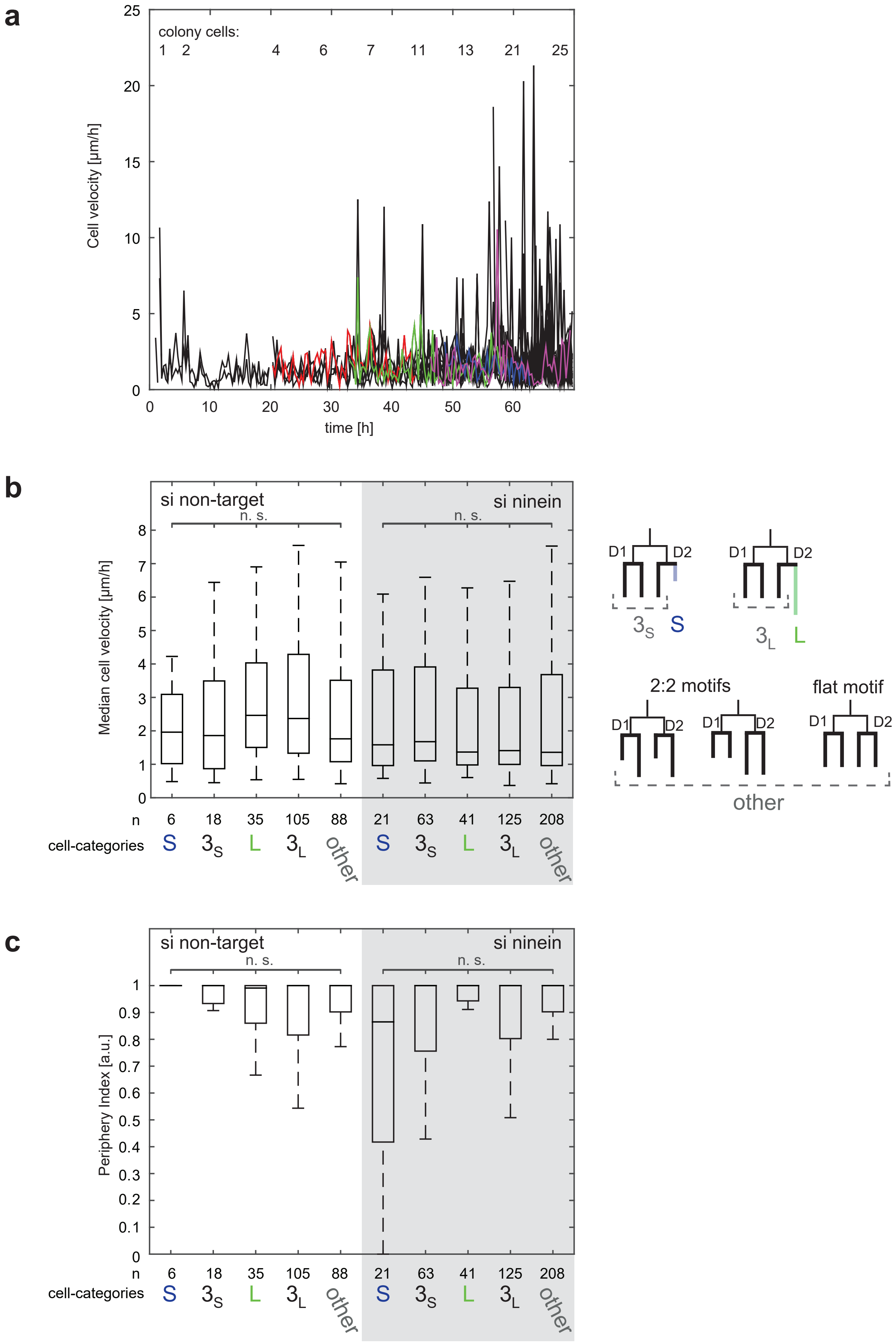

Supplementary Figure 10: Positional effects correlating with the cell classes are not detected.

a) Velocity of individual cells of the colony of Figure 3b over time. The velocities of L-cells appear similar to that of the other cells present at the same time in the colony. Colour coded are the 4 different L cells: red is the L-cell of group I, green the L-cell of group II, magenta the L-cell of group III and blue the L-cell of group IV.

b) Cell categories cannot be characterized by a specific median cell velocity for si non-target and si ninein cells. Boxplots: central mark – median; edges of horizontal box – 1st and 3rd quartiles; whiskers - 1.5 times the IQR. Right: illustration of the cell categories. Si ninein data on grey background.

c) Cell categories do not correlate with a specific position within colonies of si non-target and si ninein cells. Boxplots as in b). Si ninein data on grey background. Cell categories illustrated in b).

Supplementary Figure 11

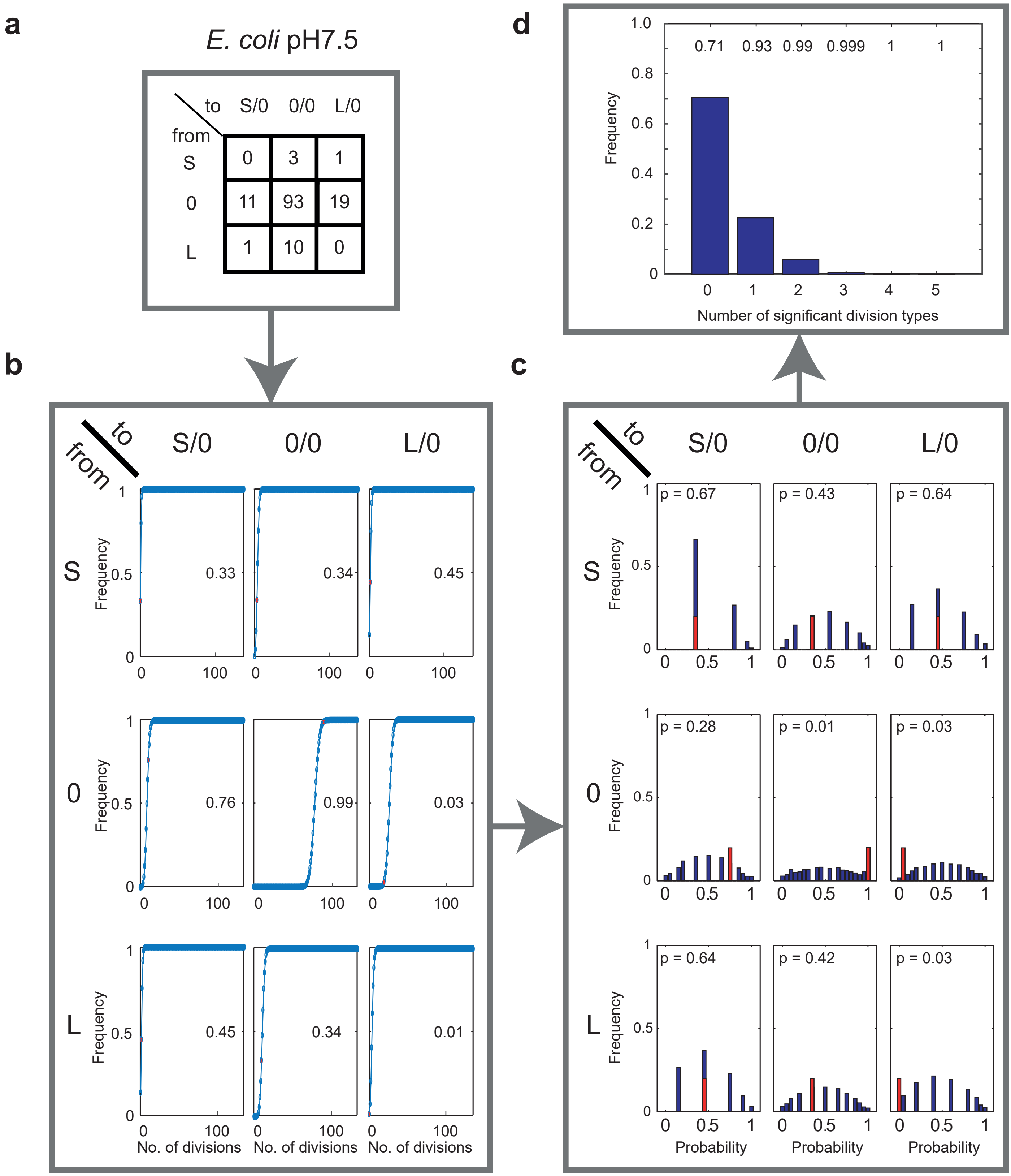

Supplementary Figure 11: Statistical propagation test for *E. coli* cells at pH7.5.

a) - d) Corresponds to description in Figure 3b-e but here for *E. coli* cells at pH7.5. The relative summary matrix is presented in Figure 5b.

Supplementary Figure 12

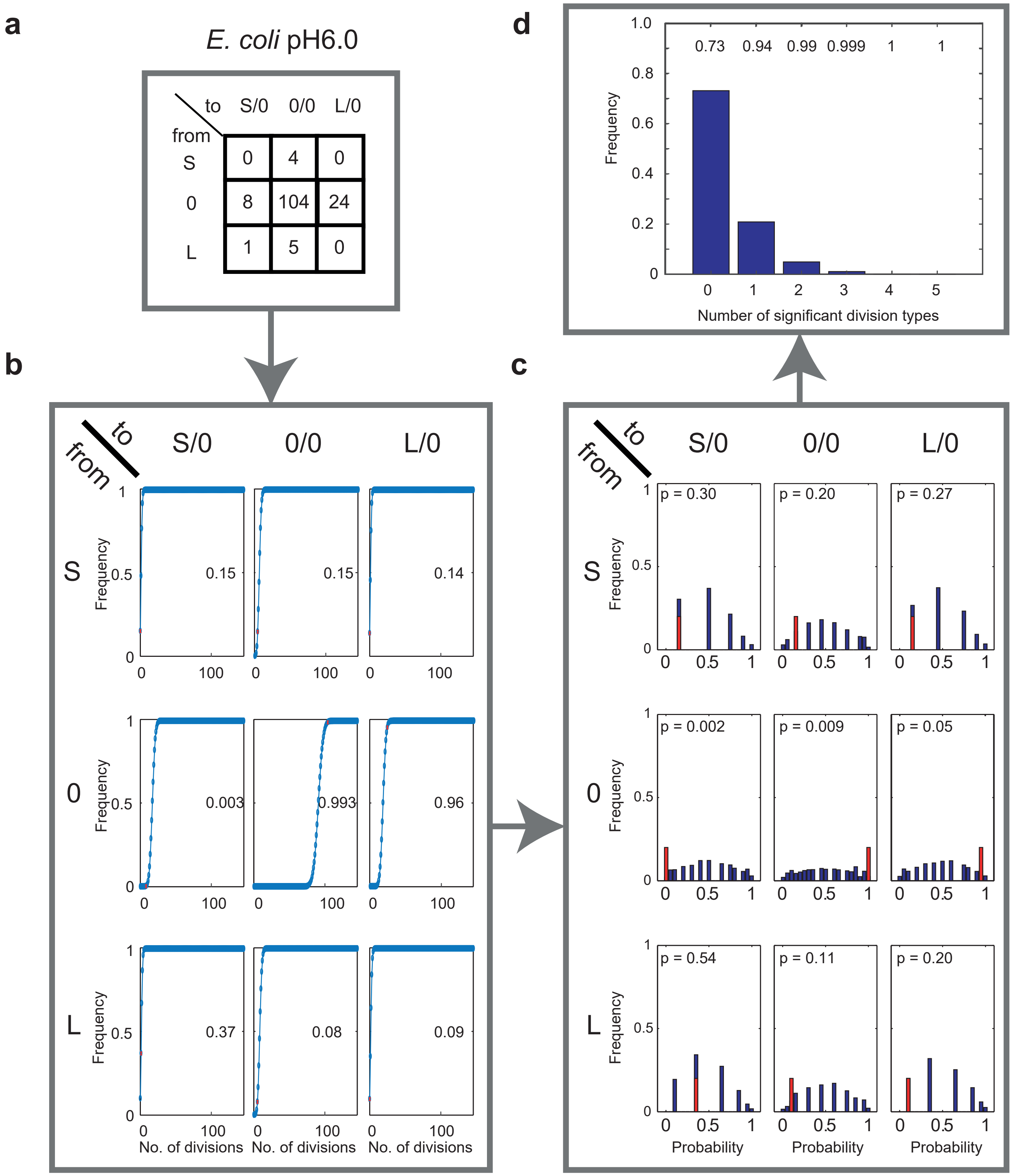

Supplementary Figure 12: Statistical propagation test for *E. coli* cells at pH6.0.

a) - d) Corresponds to description in Figure 3b-e but here for *E. coli* cells at pH6.0. The relative summary matrix is presented in Figure 5b.

Supplementary Figure 13

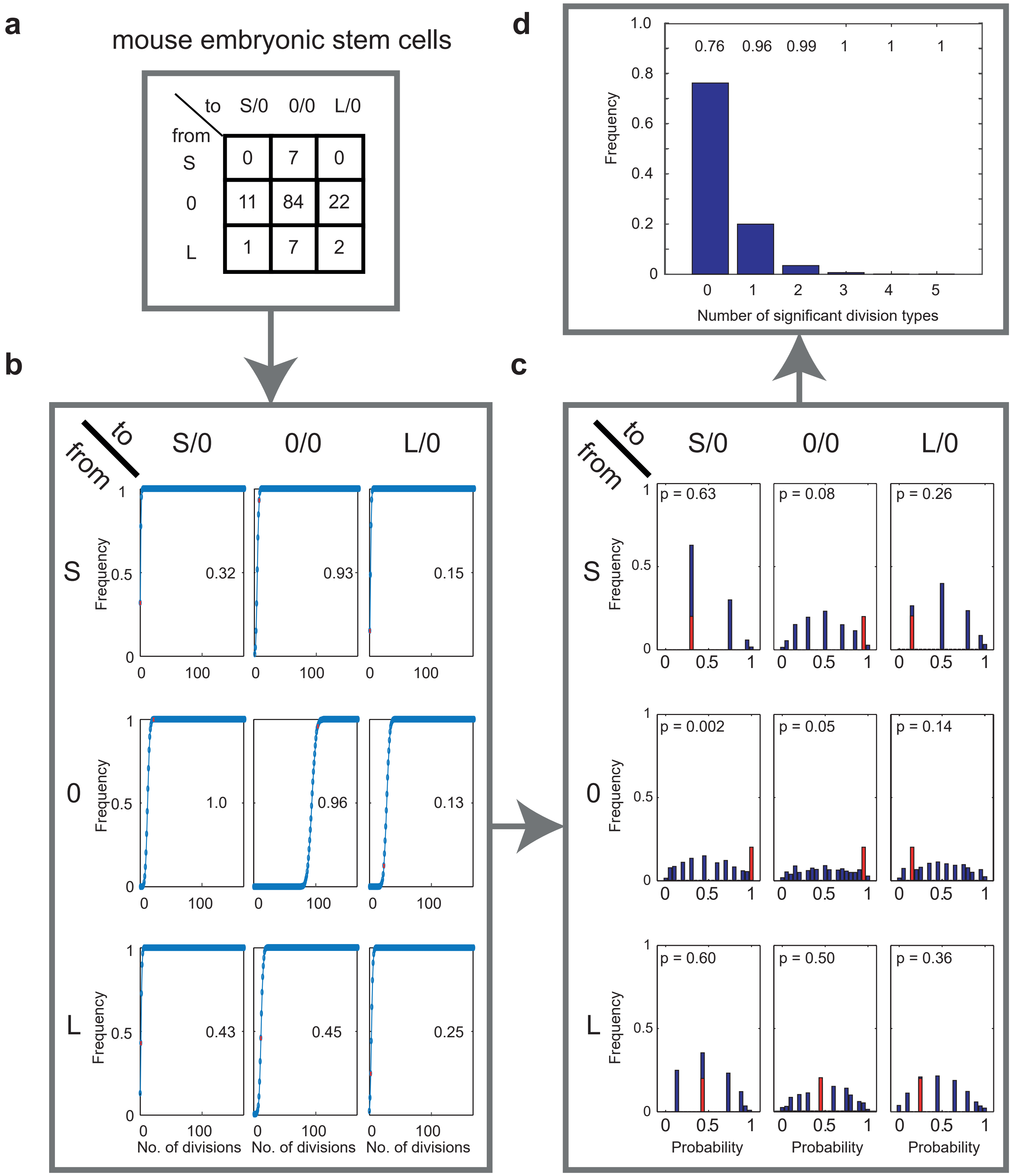

Supplementary Figure 13: Statistical propagation test for mouse embryonic stem cells.

a) - d) Corresponds to description in Figure 3b-e but here for mouse embryonic stem cells. The relative summary matrix is presented in Figure 5c.

Supplementary Figure 14

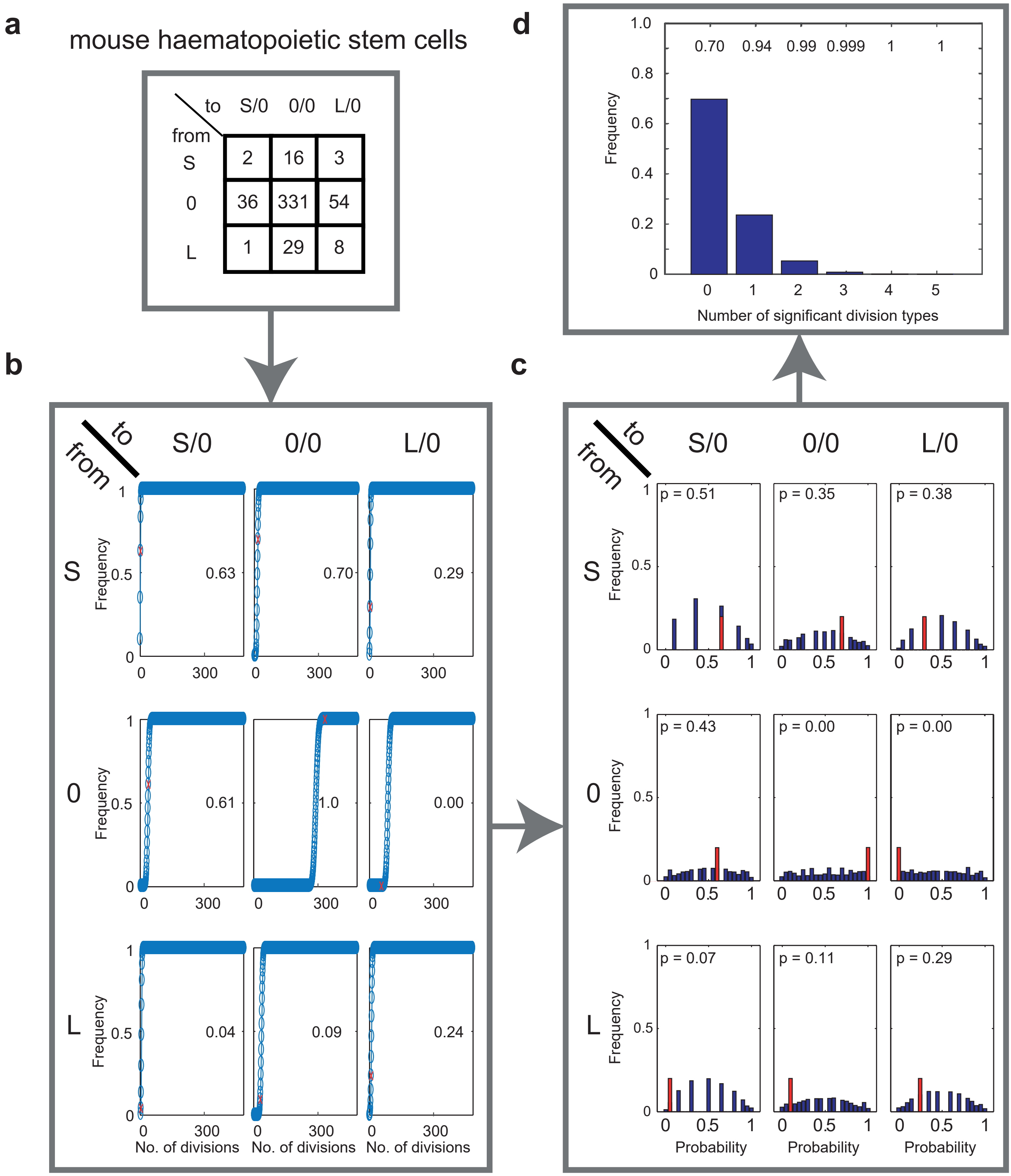

Supplementary Figure 14: Statistical propagation test for mouse haematopoietic stem cells.

a) - d) Corresponds to description in Figure 3b-e but here for mouse haematopoietic stem cells. The relative summary matrix is presented in Figure 5d.

Supplementary Figure 15

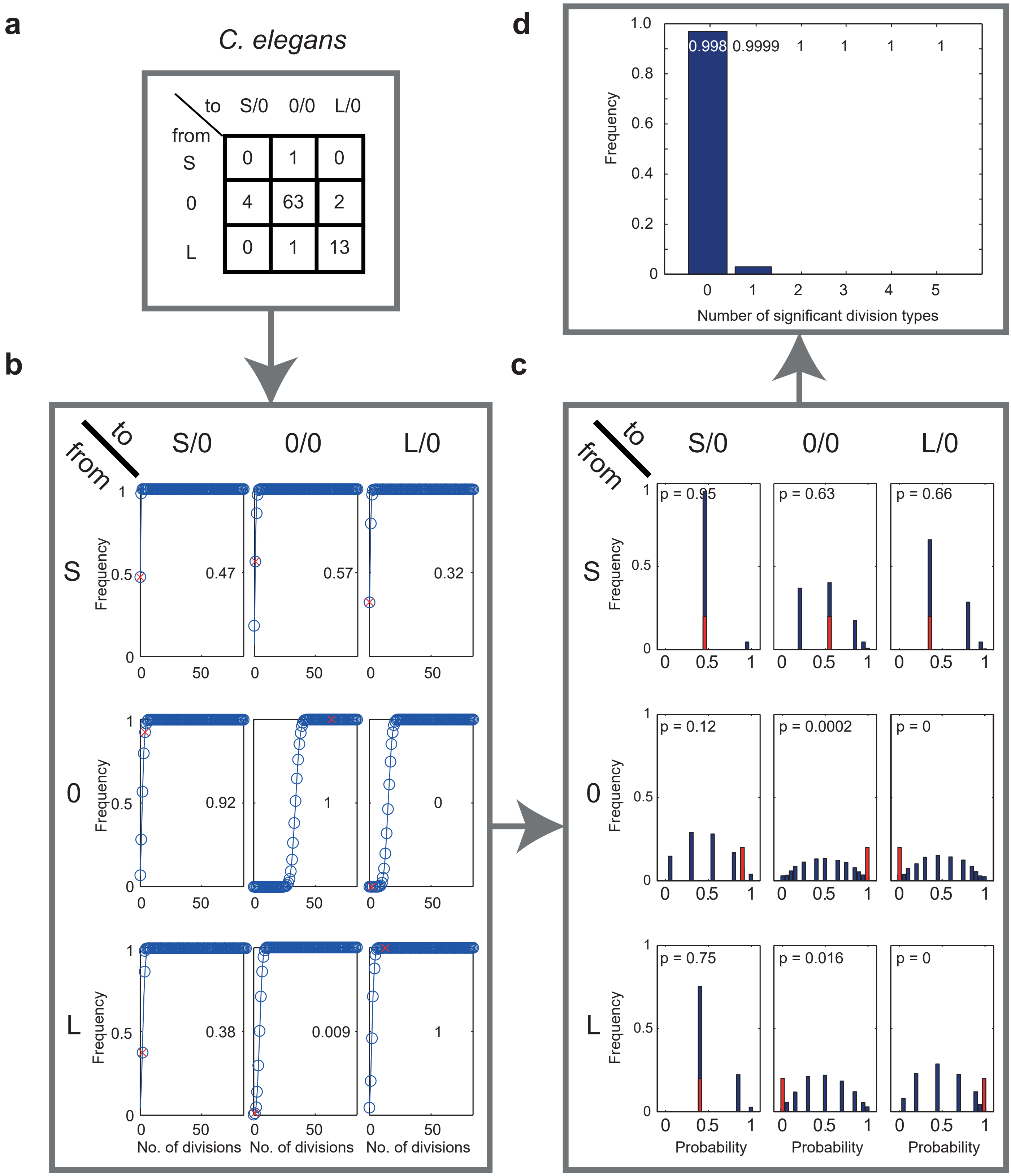

Supplementary Figure 15: Statistical propagation test for the P1-cell progeny of *C. elegans* based on 7 lineages up to generation 5.

a) - d) Corresponds to description in Figure 3b-e but here for the P1-cell progeny of *C. elegans*. The relative summary matrix is presented in Figure 5e.

Supplementary Figure 16

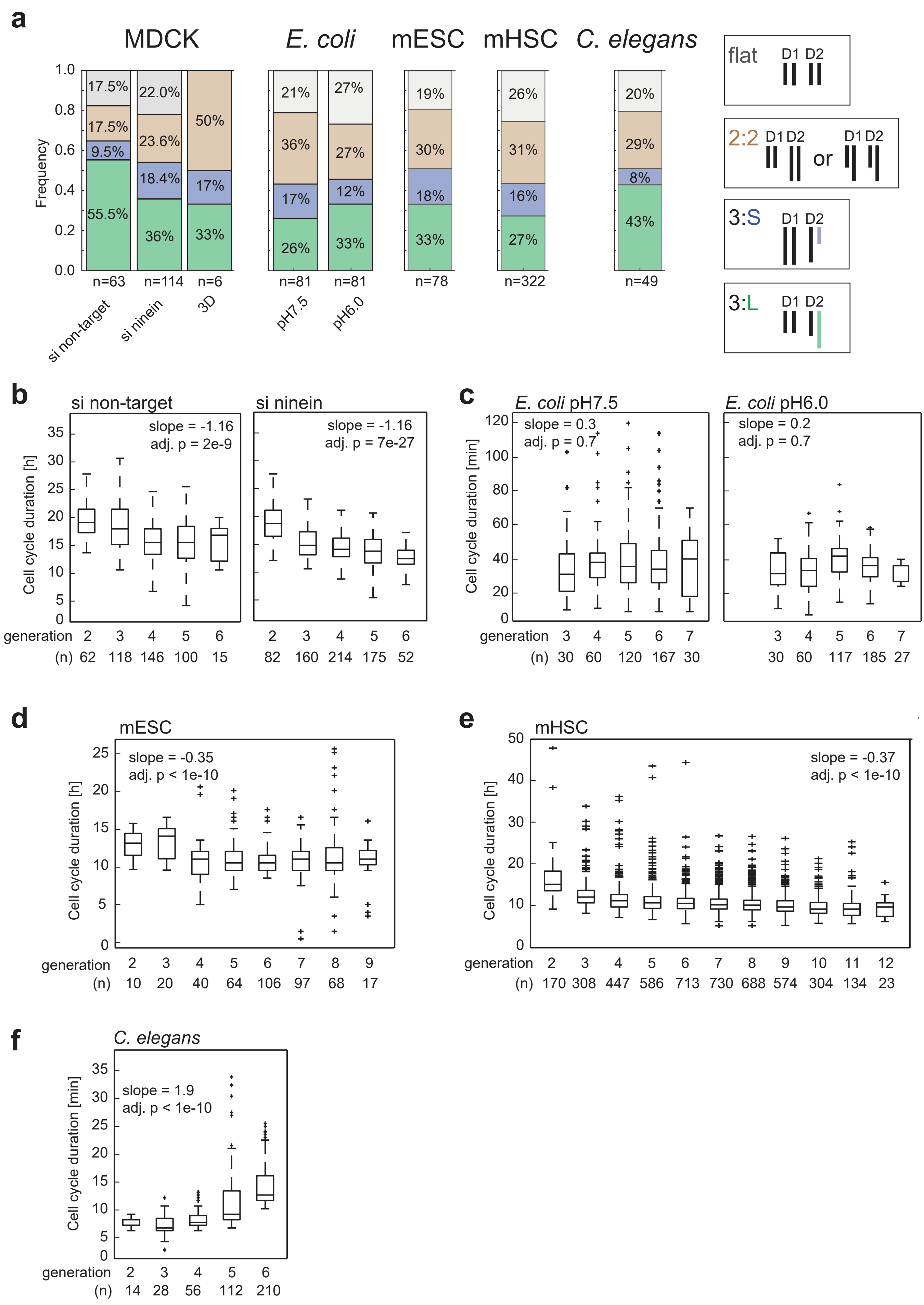

Supplementary Figure 16: Comparisons of motif frequencies and cell cycle duration distributions in 5 model systems.

a) The frequency of granddaughter sets exhibiting the 3:L- (green), 3:S- (blue), 2:2 (brown), and flat (grey) motif based on the definition in Figure 2 (threshold 2). Stacked bar plots from left to right: MDCK cells (si non-target, si ninein, 3D (non-transfected and grown in 3-dimensional extracellular matrix)), *E. coli* (pH7.5, pH6.0), mouse embryonic stem cells (mESC), mouse haematopoietic stem cells (mHSC), and *C. elegans* (up to generation 5); n - number of analysed granddaughter sets. Right legend: motif illustration as in Figure 2a.

b) - f) Distributions of cell cycle durations per generation in MDCK cells (si non-target, si ninein) (b), *E. coli* (pH7.5, pH6.0) (c), mouse embryonic stem cells (mESC) (d), mouse haematopoietic stem cells (mHSC) (e), and *C. elegans* (f). The slope was estimated by fitting a robust linear regression. Boxplots: central mark – median; edges of horizontal box – 1st and 3rd quartiles; whiskers - 1.5 times the IQR; adj. p-value; adjusted p-value; n, number of cell cycle durations.

Supplementary Figure 17

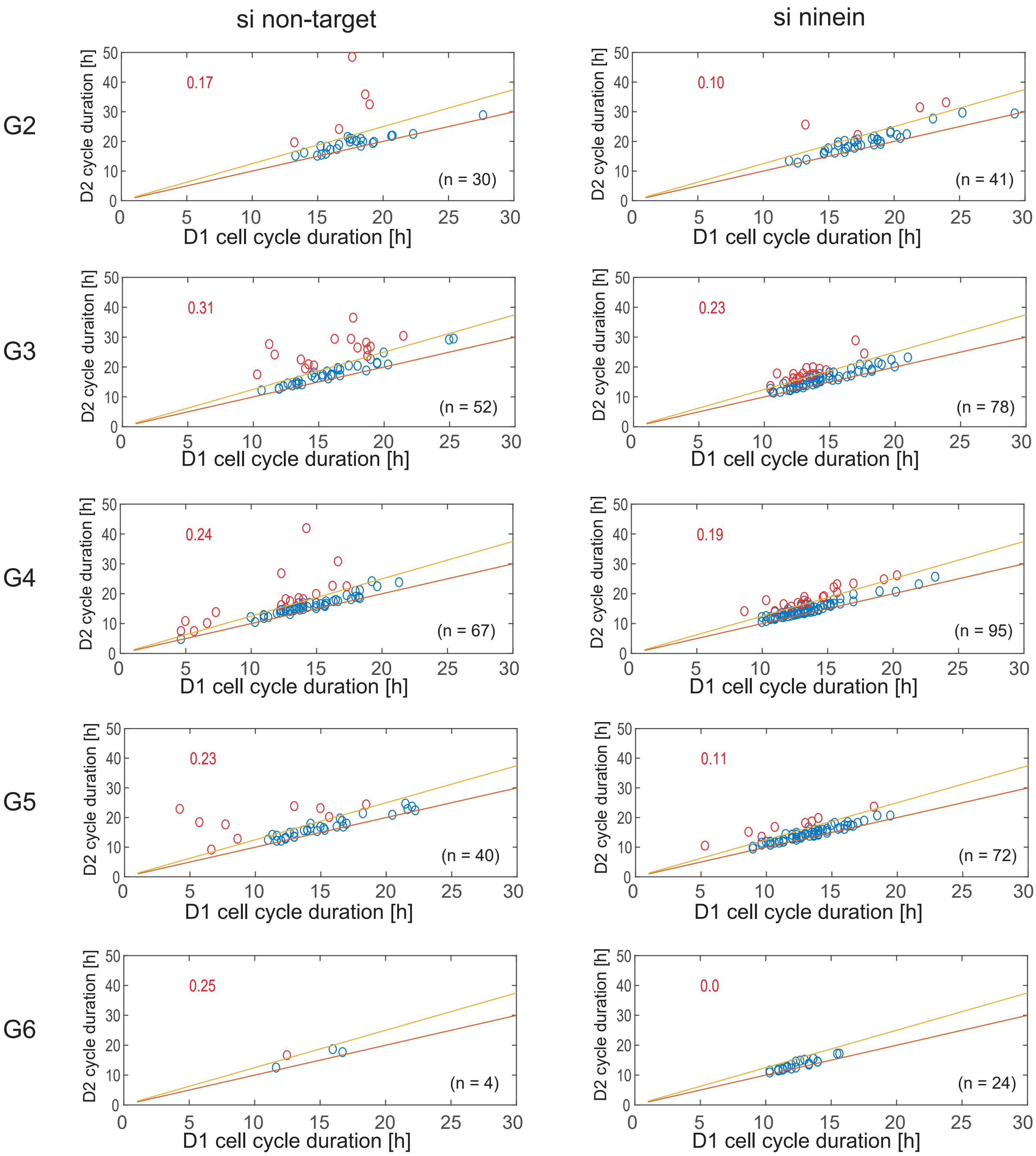

Supplementary Figure 17: More frequent and stronger differences in cell cycle durations of daughter-daughter pairs in the si non-target compared to si ninein MDCK lineages.

The cell cycle durations of all daughter-daughter pairs were analysed per generation in the lineages of the si non-target or si ninein data sets. Left: si non-target MDCK, right: si ninein MDCK. Top to bottom: Generation 2 (G2) to generation 6 (G6); G, generation. X- and y-axis, cell cycle durations of daughter 1 (D1) and daughter 2 (D2), respectively, whereby D1 has an equal or shorter cell cycle duration compared to D2. Red line: x=y; orange line: x=1.25 times y; red circles represent daughter-daughter pairs above the orange line, i.e. the cell cycle durations of those daughter pairs differ by more than 25%. Red numbers in the plots, frequency of the daughter-daughter pairs whose cell cycle durations differ more than 25%; n, number of daughter-daughter pairs.

Supplementary Tables

Supplementary Table 1

	E. coli		MDCK			stem cells (*in vitro* culture)		*C. elegans*
	pH 7.5	pH 6.0	si non-target	si ninein	3D	mESC	mHSC	*C. elegans* embryo (up to G5)
lineages	9	8	36	41	12	5	90	7
experiments	9	8	4	5	2	2	3	7
cell cycle durations	407	419	441	691	71	396	4678	210
granddaughter sets	81	81	63	114	6	78	322	49
divisions with outlier- and 0-cells (threshold 2)	138	146	70	148	0	134	480	84
origin of data	supplementary reference 1		this study		supplementary reference 2	supplementary reference 3	supplemenatry reference 4	this study

Supplementary Table 1: Origin and summary of the data of all cell systems analysed in this study. The individual numbers reflect the frequencies of the respective category mentioned in most left column. G5 – generation 5.

Supplementary Table 2

	division types	S to S/0	S to 0/0	S to L/0	0 to S/0	0 to 0/0	0 to L/0	L to S/0	L to 0/0	L to L/0
MDCK	si non-target	NaN	0	NaN	3	0.5	3	1	1	2
	si ninein	NaN	0	1	0.5	2.75	1	0	3	0
E. coli	pH7.5	NaN	1	0	1	4	3.5	0	0.5	3
	pH6.0	1	1	1	4.5	3.25	3	0	1.5	1
mESC		NaN	4	1	1	0.86*	1	0	1.5	1
mHSC		0	1	1	0.43*	6	6.4	3	2	1
C. elegans		NaN	0	NaN	2	14.5	6.5	NaN	4	11

Supplementary Table 2: Effect sizes reported as estimated robust z-scores (Supplementary Reference 1) are displayed for the individual division types in the indicated model systems and conditions, the relevant empirical number of divisions are reported in Table 1 (divisions with outlier and 0-cells (threshold 2)). For better orientation, coloured fields repeat the schematic summary of the propagation test results of Figure 5a-e. Dark grey background groups divisions of S-cells; light grey background groups divisions of L-cells; NaN, not a number (when denominator was zero); *, rounded number.

Supplementary References

1 Clark, M. W. *et al.* Periplasmic Acid Stress Increases Cell Division Asymmetry (Polar Aging) of Escherichia coli. *PLoS ONE* **10**, e0144650, doi:10.1371/journal.pone.0144650 (2015).

2 Pampaloni, F. *et al.* Tissue-culture light sheet fluorescence microscopy (TC-LSFM) allows long-term imaging of three-dimensional cell cultures under controlled conditions. *Integrative Biology* **6**, 988-998 (2014).

3 Filipczyk, A. *et al.* Network plasticity of pluripotency transcription factors in embryonic stem cells. *Nat. Cell Biol.* **17**, 1235-1246 (2015).

4 Hoppe, P. S. *et al.* Early myeloid lineage choice is not initiated by random PU.1 to GATA1 protein ratios. *Nature* **535**, 299-302 (2016).

5 Birmingham, A. *et al.* Statistical methods for analysis of high-throughput RNA interference screens. *Nat. Methods* **6**, 569-575 (2009).
