# Supplementary material for: Asymmetric division events promote variability in cell cycle duration in animal cells and Escherichia coli
Source: Nat Commun. 2019 Apr 23;10:1901. doi: 10.1038/s41467-019-09413-5 (PMC6478688; doi:10.1038/s41467-019-09413-5)
Supplement: Supplementary file 3 — Description of Additional Supplementary Files [file 41467_2019_9413_MOESM3_ESM.pdf]

Description of Additional Supplementary Files

File Name: Supplementary Movie 1
Description: Movie showing the consecutive divisions of a si non-target treated single MDCK cell expressing YFP-tagged histone2B (side by side the fluorescence channel (left) and the tracking marks from Imaris software superimposed on the fluorescence channel (right)). Data extraction from this movie resulted in the lineage tree ID8 with its corresponding cell cycle durations and cell IDs (1 frame per second = 15 min; scale bar, 20 µm).

File Name: Supplementary Movie 2
Description: Movie showing the consecutive divisions of a si ninein treated single MDCK cell expressing YFP tagged histone2B (side by side the fluorescence channel (left) and the tracking marks from Imaris software superimposed on the fluorescence channel (right)). Data extraction from this movie resulted in the lineage tree ID16 with its corresponding cell cycle durations and cell IDs (1 frame per second = 15 min; scale bar, 30 µm).

File Name: Supplementary Data 1
Description: Refers to the dataset *E. coli* at pH7.5 (e.g. Supplementary Figure 11, Figure 5b). The data matrix (no headers present) has the following shape: 1st column: cell ID in the form of 1 is the mother cells of the two daughter cells cell11 and cell12 (the schematic is also illustrated in Figure 1a). 2nd column: real time of cell division/cell death after imaging was started. 3rd column: cell cycle duration (only if the complete cellular lifetimes could be captured; otherwise NaN). 4th column: generation the cell belongs to, as illustrated in Figure 3a. 5th to 8th column: filled with NaN (not a number). 9th column: lineage ID.

File Name: Supplementary Data 2
Description: Refers to the dataset *E. coli* at pH6.0 (e.g. Supplementary Figure 12, Figure 5b). The data matrix (no headers present) has the following shape: 1st column: cell ID in the form of 1 is the mother cells of the two daughter cells cell11 and cell12 (the schematic is also illustrated in Figure 1a). 2nd column: real time of cell division/cell death after imaging was started. 3rd column: cell cycle duration (only if the complete cellular lifetimes could be captured; otherwise NaN). 4th column: generation the cell belongs to, as illustrated in Figure 3a. 5th to 8th column: filled with NaN (not a number). 9th column: lineage ID.

File Name: Supplementary Data 3
Description: Refers to MDCK si non-target (e.g. Figures 3, 5a). The data matrix (no headers present) has the following shape: 1st column: cell ID in the form of 1 is the mother cells of the two daughter cells cell11 and cell12 (the schematic is also illustrated in Figure 1a). 2nd column: real time of cell division/cell death after imaging was started. 3rd column: cell cycle duration (only if the complete cellular lifetimes could be captured; otherwise NaN). 4th column: generation the cell belongs to, as illustrated in Figure 3a. 5th to 8th column: filled with NaN (not a number). 9th column: lineage ID.

File Name: Supplementary Data 4
Description: Refers to MDCK si ninein (e.g. Supplementary Figure 9, Figure 5a). The data matrix (no headers present) has the following shape: 1st column: cell ID in the form of 1 is the mother cells of the two daughter cells cell11 and cell12 (the schematic is also illustrated in Figure 1a). 2nd column: real time of cell division/cell death after imaging was started. 3rd column: cell cycle duration (only if the complete cellular lifetimes could be captured; otherwise NaN). 4th column: generation the cell belongs to, as illustrated in Figure 3a. 5th to 8th column: filled with NaN (not a number). 9th column: lineage ID.

File Name: Supplementary Data 5
Description: Refers to non-transfected MDCK grown in 3-dimensional extracellular matrix (e.g. Supplementary Figure 16a). The data matrix (no headers present) has the following shape: 1st column: cell ID in the form of 1 is the mother cells of the two daughter cells cell11 and cell12 (the schematic is also illustrated in Figure 1a). 2nd column: real time of cell division/cell death after imaging was started. 3rd column: cell cycle duration (only if the complete cellular lifetimes could be captured; otherwise NaN). 4th column: generation the cell belongs to, as illustrated in Figure 3a. 5th to 8th column: filled with NaN (not a number). 9th column: lineage ID.

File Name: Supplementary Data 6
Description: Refers to mouse embryonic stem cells grown in non-differentiating medium (e.g. Supplementary Figure 13, Figure 5c). The data matrix (no headers present) has the following shape: 1st column: cell ID in the form of 1 is the mother cells of the two daughter cells cell11 and cell12 (the schematic is also illustrated in Figure 1a). 2nd column: real time of cell division/cell death after imaging was started. 3rd column: cell cycle duration (only if the complete cellular lifetimes could be captured; otherwise NaN). 4th column: generation the cell belongs to, as illustrated in Figure 3a. 5th to 8th column: filled with NaN (not a number). 9th column: lineage ID.

File Name: Supplementary Data 7
Description: Refers to mouse hematopoietic stem cells grown in differentiating medium (e.g. Supplementary Figure 14, Figure 5d). The data matrix (no headers present) has the following shape: 1st column: cell ID in the form of 1 is the mother cells of the two daughter cells cell11 and cell12 (the schematic is also illustrated in Figure 1a). 2nd column: real time of cell division/cell death after imaging was started. 3rd column: cell cycle duration (only if the complete cellular lifetimes could be captured; otherwise NaN). 4th column: generation the cell belongs to, as illustrated in Figure 3a. 5th to 8th column: filled with NaN (not a number). 9th column: lineage ID.

File Name: Supplementary Data 8
Description: Refers to the developing *C. elegans* zygote up to including generation 5 (e.g. Supplementary Figure 15, Figure 5e). The data matrix (no headers present) has the following shape: 1st column: cell ID in the form of 1 is the mother cells of the two daughter cells cell11 and cell12 (the schematic is also illustrated in Figure 1a). 2nd column: real time of cell division/cell death after imaging was started. 3rd column: cell cycle duration (only if the complete cellular lifetimes could be captured; otherwise NaN). 4th column: generation the cell belongs to, as illustrated in Figure 3a. 5th to 8th column: filled with NaN (not a number). 9th column: lineage ID.
