# Supplementary material for: Asymmetric division events promote variability in cell cycle duration in animal cells and Escherichia coli
Source: Nat Commun. 2019 Apr 23;10:1901. doi: 10.1038/s41467-019-09413-5 (PMC6478688; doi:10.1038/s41467-019-09413-5)
Supplement: Supplementary file 14 — Reporting Summary [file 41467_2019_9413_MOESM14_ESM.pdf]

nature research

Corresponding author(s): Ruth Kroschewski

Last updated by author(s): 2019 03 17

Reporting Summary

Nature Research wishes to improve the reproducibility of the work that we publish. This form provides structure for consistency and transparency in reporting. For further information on Nature Research policies, see Authors & Referees and the Editorial Policy Checklist.

Statistics

For all statistical analyses, confirm that the following items are present in the figure legend, table legend, main text, or Methods section.

n/a	Confirmed	
☐	☒	The exact sample size (*n*) for each experimental group/condition, given as a discrete number and unit of measurement
☐	☒	A statement on whether measurements were taken from distinct samples or whether the same sample was measured repeatedly
☐	☒	The statistical test(s) used AND whether they are one- or two-sided<br>*Only common tests should be described solely by name; describe more complex techniques in the Methods section.*
☐	☒	A description of all covariates tested
☐	☒	A description of any assumptions or corrections, such as tests of normality and adjustment for multiple comparisons
☐	☒	A full description of the statistical parameters including central tendency (e.g. means) or other basic estimates (e.g. regression coefficient) AND variation (e.g. standard deviation) or associated estimates of uncertainty (e.g. confidence intervals)
☐	☒	For null hypothesis testing, the test statistic (e.g. *F*, *t*, *r*) with confidence intervals, effect sizes, degrees of freedom and *P* value noted<br>*Give P values as exact values whenever suitable.*
☒	☐	For Bayesian analysis, information on the choice of priors and Markov chain Monte Carlo settings
☐	☒	For hierarchical and complex designs, identification of the appropriate level for tests and full reporting of outcomes
☐	☒	Estimates of effect sizes (e.g. Cohen's *d*, Pearson's *r*), indicating how they were calculated

Our web collection on statistics for biologists contains articles on many of the points above.

Software and code

Policy information about availability of computer code

Data collection	MDCK si treated: MetaMorph Version 7.5.6; Imaris 7.1.1 (Bitplane, Schlieren, Switzerland)<br>Lineage tree data established for this study: MDCK: Pampaloni, F. et al. Tissue-culture light sheet fluorescence microscopy (TC-LSFM) allows long-term imaging of three-dimensional cell cultures under controlled conditions. Integrative biology: quantitative biosciences from nano to macro 6, 988-998, (2014). C. elegans: Schnabel, R. et al. Assessing normal embryogenesis in Caenorhabditis elegans using a 4D microscope: variability of development and regional specification. Developmental Biology 184, 234-265, (1997).<br>Lineage tree data collection information of published data used in this study can be found in: mHSC: Hoppe, P. S. et al. Early myeloid lineage choice is not initiated by random PU.1 to GATA1 protein ratios. Nature 535, 299-302, (2016). mESC: Filipczyk, A. et al. Network plasticity of pluripotency transcription factors in embryonic stem cells. Nature Cell Biology 17, 1235-1246, (2015). E. coli: 1. Clark, M. W. et al. Periplasmic Acid Stress Increases Cell Division Asymmetry (Polar Aging) of Escherichia coli. PLoS ONE 10, e0144650, (2015)
Data analysis	commercial: MATLAB R2016b, self-made: Propagation Analysis Code version 1.0 (see: https://github.com/celldiversitylab/Propagation_Analysis_Code)

For manuscripts utilizing custom algorithms or software that are central to the research but not yet described in published literature, software must be made available to editors/reviewers. We strongly encourage code deposition in a community repository (e.g. GitHub). See the Nature Research guidelines for submitting code & software for further information.

Data

Policy information about availability of data

All manuscripts must include a data availability statement. This statement should provide the following information, where applicable:

- Accession codes, unique identifiers, or web links for publicly available datasets
- A list of figures that have associated raw data
- A description of any restrictions on data availability

The source data underlying Figures 1-5 and Supplementary Figures 1, 3-4, 6, 8-17 are provided as Supplementary Data 1-8. Other data that support the findings of this study is available upon reasonable request for si non-target and si ninein MDCK tracked lineages from R. Kroschewski (ruth.kroschewski@bc.biol.ethz.ch), for E.

coli lineages 28 from Joan Slonczewski (slonczewski@kenyon.edu), for C. elegans lineages from R. Schnabel (r.schnabel@tu-bs.de), for mESC 29 and mHSC lineages 30 from T. Schroeder (timm.schroeder@bsse.ethz.ch).

Field-specific reporting

Please select the one below that is the best fit for your research. If you are not sure, read the appropriate sections before making your selection.

☒ Life sciences ☐ Behavioural & social sciences ☐ Ecological, evolutionary & environmental sciences

For a reference copy of the document with all sections, see nature.com/documents/nr-reporting-summary-flat.pdf

Life sciences study design

All studies must disclose on these points even when the disclosure is negative.

Sample size	No sample-size calculation was done. The sample sizes were given by the available tracked lineage trees and for si treated MDCK cells by the number of tracable cell-colonies in more than 3 experiments per condition.
Data exclusions	No data was excluded for the diverse model systems but for one model system. Here, out of 101 blindly picked lineages of the complete published data set of the differentiating haematopoietic stem cell lineages (Hoppe, P. S. et al. Early myeloid lineage choice is not initiated by random PU.1 to GATA1 protein ratios. Nature 535, 299-302, (2016)) 30 those lineages containing cells with the 25 shortest cell cycle durations in the 101 lineage data set were censored, as these likely represent tracking errors. The resulting 90 differentiating haematopoietic stem cell lineages are the basis for the here presented results.
Replication	The discovery of 3:1 outlier motifs; these motifs are present in 3D cultured MDCK cells, si non-target (4 independent experiments) and si ninein (5 independent experiments) treated MDCK cells, E. coli (ph7.5 and pH6), C. elegans, mHSC, mESC. Discovery of non-stochastic propagation matrices composed of outlier cells; these are present in si non-target MDCK cells, E. coli pH6.0, mHSC and C. elegans.
Randomization	For cell culture irrelevant: Single MDCK cells were seeded into two culture dishes. One dish was treated with si ninein, the other dish was treated with si non-target RNA. For Figure 1c data was sub-sampled of 60 cell pairs. For propagation tests and threshold scans in Figures 2c,d, 3, 4a, 5 a-e, S8, S9, S11- S15 cell cycle durations were permuted within the same generation across all lineage trees of one model system and condition.
Blinding	Without knowing the condition (si ninein or si non-target) several investigators evaluated independently individual periods of MDCK movies to assign per imaged time point the

Reporting for specific materials, systems and methods

We require information from authors about some types of materials, experimental systems and methods used in many studies. Here, indicate whether each material, system or method listed is relevant to your study. If you are not sure if a list item applies to your research, read the appropriate section before selecting a response.

Materials & experimental systems

n/a	Involved in the study	
☐	☒	Antibodies
☐	☒	Eukaryotic cell lines
☒	☐	Palaeontology
☒	☐	Animals and other organisms
☒	☐	Human research participants
☒	☐	Clinical data

Methods

n/a	Involved in the study	
☒	☐	ChIP-seq
☒	☐	Flow cytometry
☒	☐	MRI-based neuroimaging

Antibodies

Antibodies used	anti-γ-tubulin (Sigma (order nr:T6557) ms 1:5000), anti-IgG secondary rabbit (Invitrogen (A11034) gt 1:500), and anti-IgG mouse (Invitrogen (A21236) gt 1:500) antibodies labeled with Alexa Fluor dyes. Rabbit anti-Ninein (1:1000) was a kind gift from Michel Bornens (Institute Curie, Paris, France; described in: Bouckson-Castaing, V. et al. Molecular characterisation of ninein, a new coiled-coil protein of the centrosome. Journal of Cell Science 109 (Pt 1), 179-190, (1996).
Validation	anti-γ-tubulin: immunofluorescence, westernblot, many other publications; A11034: Immunofluoresecence, minimised cross-reactivity, the goat anti-rabbit IgG whole antibodies have been highly cross-adsorbed against bovine IgG, goat IgG, mouse IgG, rat IgG, and human IgG; A21236: immunofluorescence, cross adsorption against bovine IgG, goat IgG, rabbit IgG, rat IgG, human IgG and human serum; anti ninein: immunofluorescence "The specificity of labelling was assessed by including a 20-fold molar excess of the competing peptide, Pep3, in the primary antibody"from: Journal of Cell Science 109 (Pt 1), 179-190, (1996).

Eukaryotic cell lines

Policy information about cell lines

Cell line source(s)	MDCK cells (source: I. Mellman laboratory (when at Yale University, USA); mESC: Filipczyk, A. et al. Network plasticity of pluripotency transcription factors in embryonic stem cells. Nature Cell Biology 17, 1235-1246, (2015). mHSC: Hoppe, P. S. et al. Early myeloid lineage choice is not initiated by random PU.1 to GATA1 protein ratios. Nature 535, 299-302, (2016).
Authentication	MDCK: 16SRNA fragment amplification as authentication method.<br>Authentication method published: mHSC: Hoppe, P. S. et al. Early myeloid lineage choice is not initiated by random PU.1 to GATA1 protein ratios. Nature 535, 299-302, (2016). mESC: Filipczyk, A. et al. Network plasticity of pluripotency transcription factors in embryonic stem cells. Nature Cell Biology 17, 1235-1246, (2015).
Mycoplasma contamination	Cells were tested mycoplasma-free before and after the experiments.
Commonly misidentified lines<br>(See ICLAC register)	MDCK is not listed in the ICLAC database v8.0
